# Sub-threshold depolarizing pre-pulses can enhance the efficiency of biphasic stimuli in transcutaneous neuromuscular electrical stimulation

**DOI:** 10.1007/s11517-018-1851-y

**Published:** 2018-06-09

**Authors:** Jose Luis Vargas Luna, Winfried Mayr, Jorge-Armando Cortés-Ramirez

**Affiliations:** 1 Center for Medical Physics and Biomedical Engineering, Medical University of Vienna, Währinger Gürtel 18-20, 1090 Vienna, Austria; 2 Tecnologico de Monterrey, Escuela de Ingeniería y Ciencias, Ave. Eugenio Garza Sada 2501 Sur, 64849 Monterrey, Mexico

**Keywords:** Electrical stimulation, Depolarizing pre-pulse, Conditioning, M-wave, H-reflex

## Abstract

There is multiple evidence in the literature that a sub-threshold pre-pulse, delivered immediately prior to an electrical stimulation pulse, can alter the activation threshold of nerve fibers and motor unit recruitment characteristics. So far, previously published works combined monophasic stimuli with sub-threshold depolarizing pre-pulses (DPPs) with inconsistent findings—in some studies, the DPPs decreased the activation threshold, while in others it was increased. This work aimed to evaluate the effect of DPPs during biphasic transcutaneous electrical stimulation and to study the possible mechanism underlying those differences. Sub-threshold DPPs between 0.5 and 15 ms immediately followed by biphasic or monophasic pulses were administered to the tibial nerve; the electrophysiological muscular responses (motor-wave, M-wave) were monitored via electromyogram (EMG) recording from the soleus muscle. The data show that, under the specific studied conditions, DPPs tend to lower the threshold for nerve fiber activation rather than elevating it. DPPs with the same polarity as the leading phase of biphasic stimuli are more effective to increase the sensitivity. This work assesses for the first time the effect of DPPs on biphasic pulses, which are required to achieve charge-balanced stimulation, and it provides guidance on the effect of polarity and intensity to take full advantage of this feature.

**Graphical abstract:**
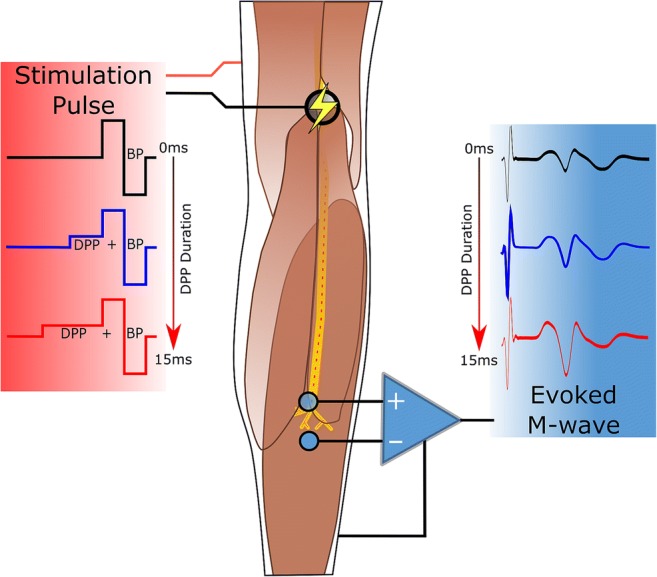
In this work, the effect of sub-threshold depolarizing pre-pulses (DPP) is investigated in a setup with transcutaneous electrical stimulation. We found that, within the tested 0–15 ms DPP duration range, the DPPs administered immediately before biphasic pulses proportionally increase the nerve excitability as visible in the M-waves recorded from the soleus muscle. Interestingly, these findings oppose published results, where DPPs, administered immediately before monophasic stimuli via implanted electrodes, led to decrease of nerve excitability.

In this work, the effect of sub-threshold depolarizing pre-pulses (DPP) is investigated in a setup with transcutaneous electrical stimulation. We found that, within the tested 0–15 ms DPP duration range, the DPPs administered immediately before biphasic pulses proportionally increase the nerve excitability as visible in the M-waves recorded from the soleus muscle. Interestingly, these findings oppose published results, where DPPs, administered immediately before monophasic stimuli via implanted electrodes, led to decrease of nerve excitability.

## Introduction

Clinical and research environments use electrical stimulation (ES) widely. ES induces an artificial electrical field that depolarizes the nerve fibers near the active electrodes and, if the depolarization is large enough, it evokes action potentials in such fibers [1]. Applied transcutaneously, ES can serve as a powerful tool for restoration of movement by non-invasive treatments and neuroprostheses applications, reducing potential risks as associated with implant-based invasive solutions.

A significant limitation of electrical stimulation is the difficulty to selectively activate target population fibers without trigger surrounding unwanted populations [2]. One approach towards increasing selectivity is the use of modified pulse waveforms, e.g., to add a sub-threshold conditioning pre-pulse before delivering a stimulating pulse [2–6]. In a series of studies with theoretical models and experiments with implanted electrodes, Grill and Mortimer presented the effects of depolarizing (DPPs) and hyperpolarizing pre-pulses (HPPs), where DPPs and HPPs tended to reduce and to increase nerve fiber excitability, respectively [2, 3, 7]. The literature on the pre-pulses application in transcutaneous setups appears controversial, in part in-line with Grill’s and Mortimer’s observations [8, 9], whereas others come to opposite conclusions [4, 10].

Studies with ex vivo experiments [7], implanted electrodes [3] and computer simulations [3, 5] have given substantial evidence that DPPs inhibit activation of the larger diameter fibers as well as fibers in close distance to stimulation electrodes. DPPs of at least 500 μs are enough to elicit the inhibitory effect, which increases proportionally to the pre-pulse duration, as tested up to 1000 μs [3]. Grill and Mortimer proposed that the non-linear properties of the cell membrane and the kinematics of the activation and inactivation gates, which control the ion channels, can explain the observed effects of the DPPs [2]. This feature may result useful to invert the natural recruiting order of electrical stimulation—fibers with large diameters and close distances to the electrodes are recruited before smaller and more distant fibers—allowing to selectively activate smaller fatigue-resistant fibers and activate distant fibers without activating fibers in the closest areas to the electrodes. In later reports on in vitro experiments with saphenous nerves from adult rats, 300-ms-long ramp-shaped DPPs with 10–40% of the original threshold intensity lowered the threshold for 1 ms monophasic stimuli, whereas higher DPP intensity elevated it [11]. Similar observations have been pointed out in transcutaneous stimulation of the median nerve in humans, where the activation threshold of the abductor *pollicis brevis* muscle was reduced with ramped DPPs of 100 ms duration and intensities below 40% of the excitation threshold, while higher intensities of the same DPPs increased the threshold [4].

Only a few publications are available on the effect of DPPs on transcutaneous ES, and those have vastly different setups and findings. Elevation of pain threshold of monophasic stimuli in relation with neuromuscular activation was reported, when 10–60-ms-long DPPs were added, with electrodes placed at the fingertips [8] or upper arm [9]. On the other hand, Willand and De Bruin investigated the effect of ramp-shaped and rectangular DPPs with a duration of 5 ms on median nerve stimulation. Their single case study concluded that, contrary to predictions of computational models, DPPs of just 10% of the intensity of the active stimulation pulse, in this case with 0.1 ms duration, consistently increased the responses of the thenar muscle [10].

In literature, DPP shape varies between ramp and rectangle in a wide duration range. For Grill and Mortimer, 0.5 ms rectangular DPPs were enough to induce a conformational change on the ionic channels gates [2, 3]; Poletto and Van Doren used 10-ms-long rectangular DPPs for stimulation with cutaneous electrodes [8], and Hennings et al. tested triangular DPPs of up to 500 ms [4], observing varying effects depending on the DPP duration and intensity. In the latter case, the long duration raises two issues for clinical applications. Firstly, it limits the stimulation frequency to values that cannot induce fused muscle contractions, and secondly, the use of such long pulses may dangerously increase the chance of tissue damage due to electroporation [12, 13]. Although few clinical applications use high-intensity and long-duration (up to 500 ms) pulses, electrode size, construction, and application procedures are highly restricted due to safety regulations, e.g., for activating denervated muscles. For the application of long-duration DPPs, it is essential to comply with similar safety precautions. On the other hand, standard nerve stimuli, with typically less than 1-ms phase duration and much lower pulse charge transfer, are much less critical in this respect.

It is important to note that nearly all previous reports on the effect of DPPs on fiber excitability only have analyzed combinations with monophasic pulses, which have limitations for long-term application regarding nerve fiber recruitment efficacy and charge imbalance [14, 15]. Because the DPPs have the same polarity of the activation pulse, they increase the charge imbalance per pulse. Therefore, the use of an additional compensatory phase, to establish appropriate charge-balance, would improve practicability and safety. The projection of earlier findings on DPPs effects on monophasic stimuli to biphasic pulses is not trivial since previous reports have shown that adding a second phase and changes in the polarity sequence can substantially modify the motor response [16, 17]. On the one hand, these reports conclude that regardless the polarity sequence, the cathodic phase is always triggering the action potential. Moreover, the anodic phase could reduce the motor output either by blocking the action potential propagation (when applied at the end) or by pre-conditioning the fibers (when applied first) [17].

This work is aimed to study the effect of DPPs on biphasic stimuli administered via transcutaneous electrodes and to find explanations for the variety of inconsistent results we found in the literature. We applied transcutaneous stimulation on the tibial nerve using DPPs of different lengths, finding that the muscle responses were consistently enhanced.

## Methods

A trial study was defined to provide sufficient data to show statistical significance of the conditioning effects of DPPs on biphasic pulses. The measurement protocol was approved by the Ethics in Research Committee and the Research Committee of the School of Medicine of the Tecnológico de Monterrey (Mexico). All measurements were conducted according to the principles of Helsinki Declaration.

A total of nine volunteers (six males, three females), age between 23 and 28 years (M = 25.50, SD = 1.65), were enrolled in the protocol. The subjects were healthy people with no record of neurological diseases or dermatological problems in the stimulated and monitored area. The participants were informed about the experimental procedure and potential risks. All subjects signed an informed consent letter before the measurements.

Single current-controlled pulses were applied to the right tibial nerve via a unipolar electrode configuration to assess the effect of DPPs in evoked responses of the soleus muscle. In this setup, the neuromuscular responses due to direct excitation of α-motoneurons—also known as M-wave—and a central contribution driven by the evoked potentials in Ia afferent fibers—also known as H-reflex—have different latencies, which make them easy to identify as independent responses in the soleus electromyogram (EMG) [18]. This difference is because the induced action potentials on the motoneuron travel directly to the neuromuscular junction, while on the afferent fibers, the volleys travel to the spinal cord, where they lead to a monosynaptic response on the motoneuron and then propagate down to the innervated muscle.

The behavior of both responses—M-wave and H-reflex—is different. With a growing stimulus intensity, the M-wave amplitude grows and finally saturates, whereas the H-reflex reaches a maximum amplitude (H_max_) and then gradually attenuates until it disappears. The observed attenuation of the H-reflex is caused by antidromic action potentials in the α-motoneuron that collide with orthodromic reflex responses [18]. Because of the different behavior, the effect of the DPPs in M-waves and H-reflexes was independently evaluated. Finally, the results were critically compared with other work from literature, where only monophasic pulses have been applied.

### Stimulation protocols

**Fig. 1:**
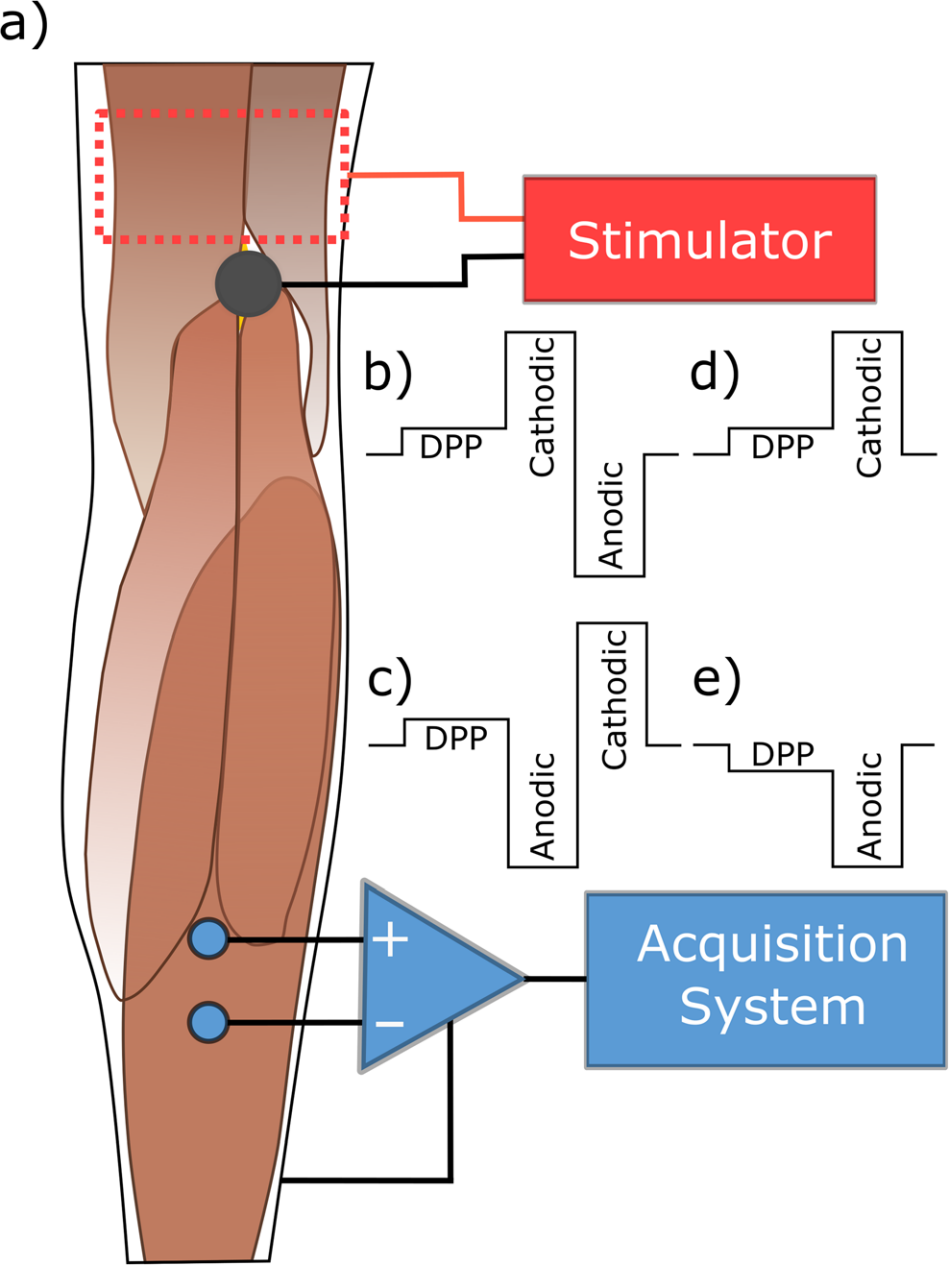
Scheme of the measurement’s setup. a) Measurement setup and electrodes placement (stimulation in black and red, and EMG in blue) for all the protocols. Depolarizing pre-pulses preceding biphasic, b) cathodic-anodic, and c) anodic-cathodic stimuli and monophasic, d) cathodic, and e) anodic stimuli with DPPs. Adapted from [19]

Electrical stimulation was applied through self-adhesive hydrogel electrodes (Hivox Biotek Inc., Taiwan). A reference electrode (5 × 10 cm) was placed on the anterior thigh (above the patella), and an active electrode (diameter 2.5 cm) was placed over the tibial nerve at the *popliteal fossa* level (Fig. 1a) [18]. The position of the active electrode was pre-assessed with a spherical stainless-steel test electrode (diameter of 2.5 cm) and electrode gel for better skin contact. The hydrogel electrode was then placed where a maximum soleus response to low-intensity (8–15 mA) biphasic pulses of 0.5 ms per phase had been observed. The current-controlled stimulation system was a STMISOLA output stage (BIOPAC Systems, Inc., USA), controlled via an NI MyDAQ (National Instruments Corp., USA).

In the first part of the protocol, rectangular charge-balanced biphasic pulses with a duration of 0.5 ms per phase and monophasic pulses of 0.5 ms were applied. The intensities of the stimulating pulses (biphasic and monophasic) were determined for each subject individually (M = 18 mA, SD = 6 mA) based on the evoked M-waves elicited by cathodic-anodic biphasic pulses of 0.5 ms and fixed between the M-wave threshold and saturation intensity. Nine DPP durations (0, 0.5, 0.75, 1, 2.5, 5, 7.5, 10, and 15 ms) were selected and tested in combination with biphasic pulses of both polarities—cathodic-anodic and anodic-cathodic. These DPP durations were selected on the basis of reports on the application of monophasic pulses [2, 4, 8, 10] and limited to durations that allow continuous stimulation with 50 Hz, i.e., below 19 ms. The sub-threshold intensity of the DPPs was set to 50% of the twitch threshold for the longest applied DPP—15 ms for the assessment of M-waves and 5 ms for assessment of the H-reflex. The conditioning amplitudes were determined manually for each subject, with an average of 2.8 mA (SD = 0.6 mA) and, to ensure statistic validity, six to ten repetitions per combination were applied.

In one subject, the same measurements were performed with 5 ms DPPs to get insights into the effect of the DPP duration on the H-reflexes in a region ahead of H_max_. Additionally, this measurement provides a comparative framework to other studies that use DPPs with monophasic pulses. For this assessment, 5 ms DPPs were administered prior to biphasic and monophasic pulses of 0.5 ms per phase and in both polarities. The intensities were independently chosen for each of the four types of stimuli (cathodic-anodic, anodic-cathodic, cathodic, and anodic) and defined as the necessary intensity to evoke H-reflexes of approximately 50% of H_max_. The intensity of the DPPs was set to 50% of the activation threshold (3 mA) of a monophasic cathodic pulse of 5 ms. All values were calculated as the average of ten repetitions per combination.

Please note that for biphasic stimuli, the depolarizing pre-pulses refer to cathodic pre-pulses, since the stimulating phase is the cathodic one (Fig. 1b, c) [16]. For monophasic pulses, the pre-pulse had the same polarity as the stimulation pulse (Fig. 1d, e).

The delivery of stimulation pulses was randomized to avoid bias due to uncontrolled factors. A pause of 8 s between pulses was used to reduce possible fatigue effects and to allow full recovery of reflex mechanisms.

### Measurement setup

The stimulation responses were monitored from the evoked potentials of the soleus muscle, which were recorded via surface EMG. Prior to the application of EMG electrodes, the skin was prepared with abrasive gel to reduce the skin-electrode interface impedance. A pair of recording electrodes was placed centrally above the muscle belly with 3-cm inter-electrode distance, while the reference electrode was placed on the anterior side of the leg, over the tibia (Fig. 1a). The EMG signals were recorded with an MP35 (BIOPAC Systems, Inc., USA) unit at 2.5 kS/s. The setup was controlled and monitored with a software interface programmed in LabView™ (National Instruments Corp., USA).

All subjects were lying in prone position during the whole session to minimize the voluntary muscle activity, which could cause undesired modulation of responses [18, 20]. For the same reasons, the measurements were performed in a quiet, low-light, and temperature-controlled room [18].

### Data analysis

The datasets were post-processed in MATLAB (The MathWorks, Inc., USA). The areas of evoked M-waves were quantified for each pulse. For each subject, the values were normalized with reference to the average area of the responses evoked by cathodic-anodic biphasic pulses.

A two-way ANOVA was conducted to compare the effects the DPP duration, polarity of the biphasic pulses, and DPP-polarity interaction on the evoked M-wave responses in the soleus muscle. Additionally, an ANOVA was performed to study the effect of short DPPs (0 to 1 ms) on anodic-cathodic biphasic pulses.

The peak-to-peak (P2P) value was used to quantify the magnitude of the H-reflex. In this case, the data were normalized for each pulse configuration, based on the average value without DPP. In analogy to the M-wave assessment, a two-way ANOVA was conducted to assess the effects of the waveform (cathodic-anodic, anodic-cathodic, cathodic, anodic) and pre-pulses (no pre-pulse, cathodic, and anodic pre-pulse) on the evoked reflexes.

Welch *t* tests were also performed to analyze specific differences in the responses. For all statistical tests, the significance level (*α*) was considered equal to 0.01.

## Results

**Fig. 2:**
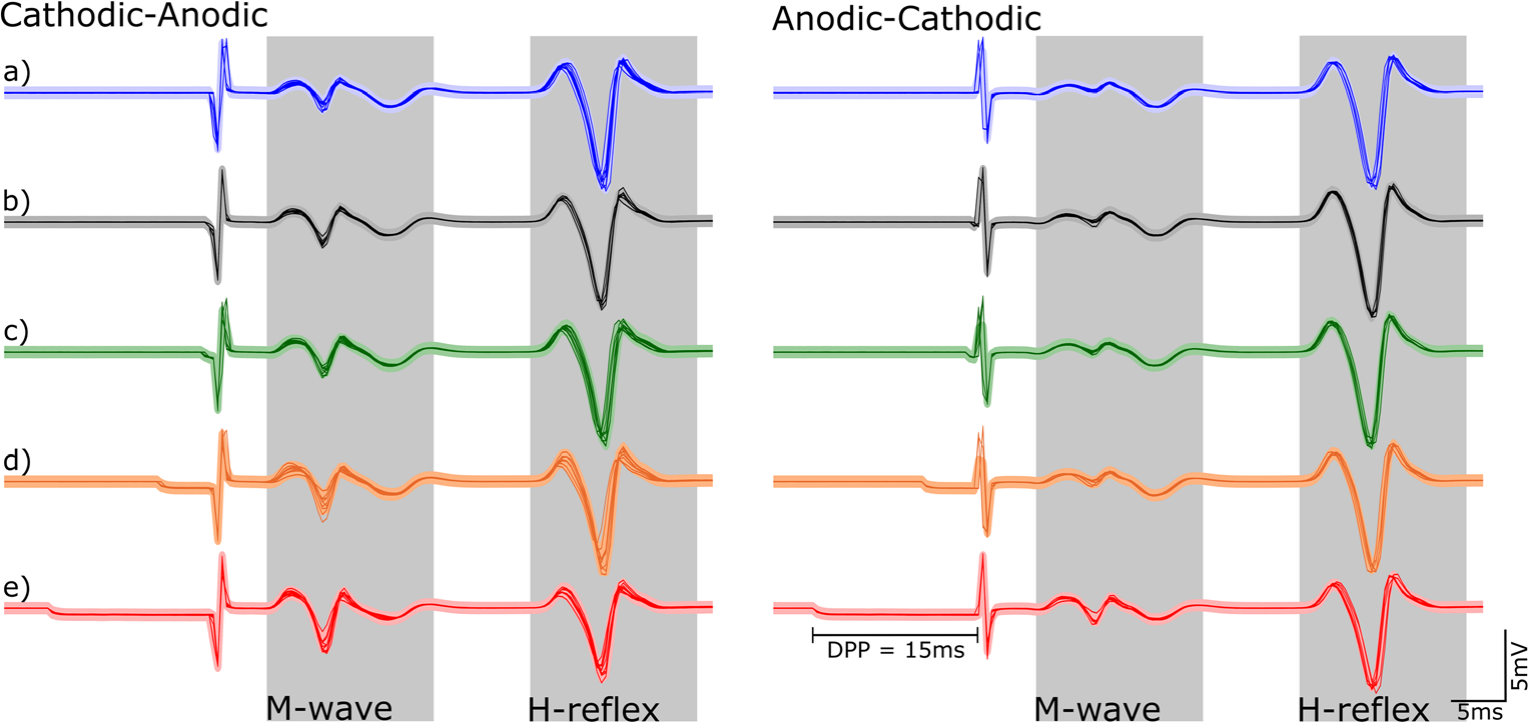
Exemplary evoked responses. Overlapped evoked potentials (*N* = 6) from subject S13. The responses were generated by biphasic pulses of both polarities with DPP durations of a) 0, b) 0.5, c) 1, d) 5, and, e) 15 ms. It is shown how the M-wave increased with the DPP duration, while the H-reflex reaches saturation and start to decrease, which follows the natural recruitment curve of such response

A total of ten valid datasets were acquired from nine volunteers. Figure 2 shows exemplary responses (*N* = 6) recorded from one subject (S13) during stimulation with different DPP durations. For the main measurements, the intensity was set to a level that elicited M-waves with a medium-range amplitude, which produced a consistent increase in the M-waves in all subjects when DPPs were added. Additionally, as shown in Fig. 2, the latencies of the M-waves remained similar regardless of the DPP duration, which suggests that the pre-pulses did not evoke any action potentials by themselves.

**Fig. 3:**
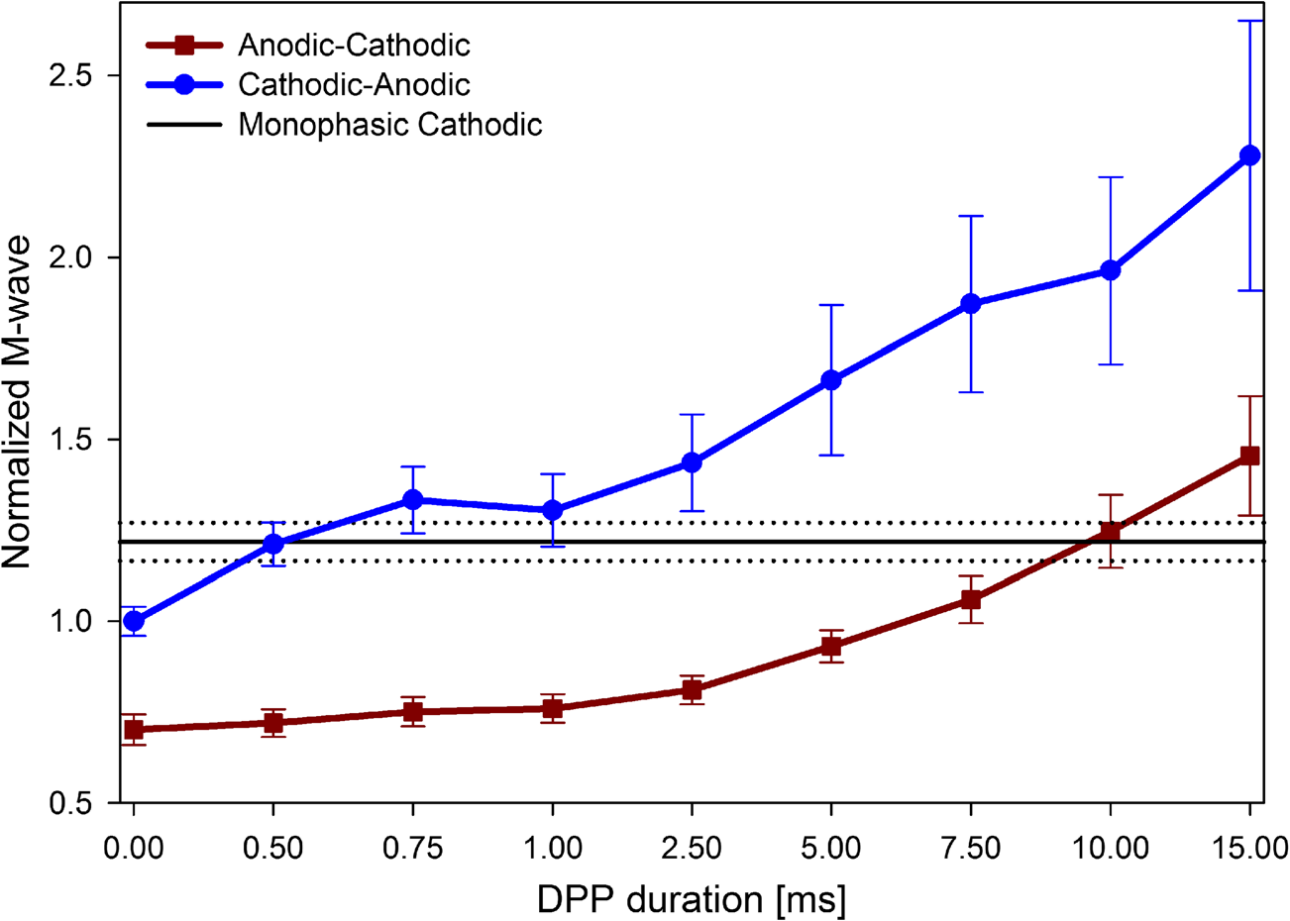
DPPs influence on evoked M-waves. Normalized mean areas (with 95% confident intervals), for all subjects, of the M-waves evoked by cathodic-anodic (circle) and, anodic-cathodic (square) biphasic pulses, when different DPP durations were applied. Horizontal lines represent the M-waves evoked by monophasic cathodic pulses. For each polarity and DPP duration *N* = 88

From the M-wave assessment, 1583 responses (including 2 polarities and 9 DPP durations) were computed. The two-way ANOVA shows a statistically significant influence of the DPP duration on the muscle response, *F* (8, 1565) = 39.25, *p* < 0.001; the polarity also has a significant effect on the evoked potentials, *F* (1, 1565) = 303.24, *p* < 0.001. The interaction between these factors, on the other hand, does not have a strong statistical effect on the stimulation outcome, *F* (8, 1565) = 2.50, *p* = 0.011. Table 1 and Fig. 3 summarize the mean of the M-wave areas (all subjects) for all tested polarities and DPP durations. The data show that the DPP duration has a strong influence in the elicited M-wave, and within the tested range, the effect does not saturate.

**Table 1:** Summary of mean (standard deviation) of normalized areas of the M-waves evoked in all subjects by biphasic pulses with different polarities and DPP durations. In all the cases *N* = 88

DPP duration (ms)	Cathodic-anodic	Anodic-cathodic
0.00	1.00 (0.19)	0.70 (0.20)
0.50	1.21 (0.28)	0.72 (0.18)
0.75	1.33 (0.44)	0.75 (0.19)
1.00	1.30 (0.48)	0.76 (0.19)
2.50	1.44 (0.64)	0.81 (0.19)
5.00	1.66 (0.99)	0.93 (0.21)
7.50	1.87 (1.16)	1.06 (0.31)
10.00	1.96 (1.23)	1.25 (0.48)
15.00	2.28 (1.78)	1.45 (0.79)

Although an enhancing effect was observed in both polarities, for anodic-cathodic pulses, the DPP-induced effect was not observed until durations higher than 1 ms. This delay is confirmed by the one-way ANOVA test on the responses of anodic-cathodic biphasic pulses with DPPs between 0 and 1 ms, which shows a non-significant influence of DPPs, *F* (3, 348) = 1.91, *p* = 0.127.

The Welch *t* test shows that, without DPPs, M-waves elicited by monophasic cathodic stimuli are consistently bigger (M = 1.23, SD = 0.29) than those following cathodic-anodic biphasic pulses (M = 1, SD = 0.19), *t* (D.F. = 161.24) = 6.35, *p* ˂ 0.001. On the other hand, sole monophasic anodic stimuli produce zero or negligible responses (M = 0.10, SD = 0.11) in comparison with cathodic-anodic biphasic pulses, *t* (D.F. = 137.50) = 38.72, *p* ˂ 0.001.

The H-reflex behavior, in response to the addition of DPPs of 5 ms, was assessed in a single case study. The results show that a DPP prior to the stimulation pulse increases the H-reflex, except by DPPs with the inverse polarity on monophasic pulses (Table 2). The ANOVA test confirmed that the stimulus waveform (*F* (3, 94) = 19.32, *p* < 0.001) and the pre-pulse (*F* (2, 94) = 35.10, *p* < 0.001) have a significant influence on the H-reflex amplitude. The data shows that, in general, when the pre-pulse has the same polarity as the stimulating phase, the peak-to-peak value of the response increases. On the other hand, cathodic pulses with an anodic pre-pulse showed a significantly smaller response, *t* (D.F. = 10.36) = 19.42, *p* = 0.001.

**Table 2:** Summary of average (standard deviation) peak-to-peak values of the H-reflexes evoked on the complementary measurement, where cathodic and anodic pre-pulses of 3 mA, and 5-ms duration were used with biphasic (BP) and monophasic (MP) stimulation. In all the cases *N* = 10

Polarity	MP cathodic—16 mA	MP anodic—30 mA	BP cathodic-anodic—18 mA	BP anodic-cathodic—19 mA
No pre-pulse	0.438 (0.063) mV	0.334 (0.077) mV	0.605 (0.036) mV	0.284 (0.071) mV
Cathodic pre-pulse	0.701 (0.015) mV	0.334 (0.032) mV	0.724 (0.009) mV	0.452 (0.155) mV
Anodic pre-pulse	0.039 (0.017) mV	0.366 (0.032) mV		

## Discussion

The use of cathodic pre-pulses in our study led to opposing observations in comparison to most of the previously published work in this field, which rather describes a reduction of the nerve fiber excitability by DPPs [2, 3, 5, 6, 8, 9]. From these studies, only two used transcutaneous stimulation, and both assessed only the pain sensation—evoked in cutaneous sensory structures—without any assessment on activation of deeper α-motoneurons or Ia afferent fibers [8, 9]. More recent work from Vastani et al. and Hennings et al. reports that “strong” DPPs effectively reduce the excitability of nerves, whereas “weak” DPPs enhance it [4, 11]. Unlike the rectangular DPPs with duration of maximally 15 ms in our protocol, those studies used ramp-shaped pre-pulses of longer durations (up to 500 ms), which is a more natural approach to accommodation and allows the use of even higher intensities for a DPP than for the intrinsic test pulse without per se reaching the activation threshold [11]. Anyhow, we can expect that a rectangular DPP with sub-threshold intensity should have similar effects on the conformational changes of the ion channels in a nerve fiber since they are driven by the electrical charge movement [21]. Presumably, the conformational changes with rectangular pre-pulses occur even at a higher speed since the voltage-dependent regions are exposed to the maximum pre-pulse intensity already from the beginning of the conditioning DPP.

Grill and Mortimer initially explained the influence of DPPs to be based on conditioning of activation and inactivation gates of the sodium channels [2]. Further electrophysiological studies have expanded the sodium channel model to consider the four domains that compose them and are gradually contributing to the activation or deactivation of the channel [21–23]. These studies show that channel activation (opening) or inactivation depends on the subset of activated domains. Armstrong presented a state model that considers the different dynamics, e.g., time constants, of the domains to explain how the energy applied activates them and influence the channel state towards open or inactive [21]. This model could explain why high-current sub-threshold DPPs can transit between resting to fully inactivated state, while low-charge DPPs are only able to activate some of the domains. In the latter case—after a DPP has activated some domains, but without reaching inactivation—the stimulating pulse requires less intensity to activate the remaining segments required for full opening of the channel. This increase in sensibility could explain, why lower stimulus intensity for triggering an action potential is required after a conditioning DPP.

If we define “strong” as higher intensity and longer duration—high electrical charge—and “weak” as short and low-intensity pulses—low electrical charge—we can use this parameter, without consideration of the waveform, for comparison and interpretation of already published results in relation to findings in our study.

All studies that have observed an excitatory effect of DPPs have been on the application of transcutaneous stimulation to depolarize subjacent nerve fibers, as in the distance of about 25 mm in our case. This implies that the current density in the tissue embedding the fibers are substantially smaller than close to the electrode surface [24]. In addition, computer simulations suggest that the spatial position of the current source in relation to the Ranvier nodes strongly affects the local transient changes in excitability by DPPs [5]. Finally, transcutaneous electrical stimulation is associated with a high voltage drop across the cutaneous and subcutaneous tissue, which has the highest electrical impedance [25]. On the other hand, on deeper nerve fibers like tibial nerve, the electrical field appears strongly attenuated or, in another word, “weaker.” In most studies that report a reduction of excitability by DPPs, the stimulation electrodes have been placed close to the targeted fibers, e.g., cuff electrodes encircling the epineurium, or transcutaneous stimulation over the cutaneous sensory nerves, which induce a local “strong” electrical field.

Interestingly, the use of short (< 1 ms) cathodic DPPs prior to anodic-cathodic biphasic pulses did not significantly influence the responses. This observation suggests that the anodic phase reduces or reverses the effect of the depolarizing pre-pulse before the cathodic phase triggers an action potential. This is consistent with the state model of Armstrong, in which the transition between the states can occur in both directions.

Finally, the application of anodic pre-pulses prior to monophasic cathodic pulses decreases the evoked neuromuscular response. This is consistent with the attenuation of the response when the polarity of a biphasic pulses is inverted from cathodic-anodic to anodic-cathodic (shown in Fig. 3). Further assessments should be done to validate such result in other electrode setups.

## Conclusions

To our knowledge, this is the first work that assesses the effect of depolarizing pre-pulses with biphasic pulses. The data show that, in transcutaneous NMES, depolarizing pre-pulses in the range of 0.5 to 15 ms enhance the activation of the nerve fibers in comparison to use of the same stimulation pulses without DPPs. Although our data suggest that the enhancing effect would increase proportionally with further increase of the DPP duration, other reports suggest that, with longer and higher intensity DPPs, the effect can change to the opposite and lead to smaller responses [4, 11].

The most pronounced enhancing effect of DPPs is accomplished if the pre-pulse has the same polarity as the stimulating phase. At least for our setup, where stimulation intensities were kept below individual pain thresholds, the stimulating phase of biphasic pulses was the cathodic one, regardless the polarity order of the phases.

The use of DPPs prior to biphasic stimuli could be applied to reduce the current intensity required for a desired neuromuscular response and, therefore, reduce potential stimulation related discomfort.
